# Rare Leukemic Phase of Follicular Lymphoma With a High-Risk Follicular Lymphoma International Prognostic Index (FLIPI) Score Treated With Bendamustine-Rituximab

**DOI:** 10.7759/cureus.109914

**Published:** 2026-05-30

**Authors:** Niyati Bansal, Steve Thomas, Sri Gayathri Shanmugam

**Affiliations:** 1 General Medicine, Sri Ramachandra Institute of Higher Education and Research, Chennai, IND; 2 Hematology, Sri Ramachandra Institute of Higher Education and Research, Chennai, IND; 3 Pathology, Sri Ramachandra Institute of Higher Education and Research, Chennai, IND

**Keywords:** bendamustine, br regimen, flipi, follicular lymphoma, leukemia, leukemic phase of follicular lymphoma, lymphoma, pod24, rituximab

## Abstract

Follicular lymphoma is a slow-growing B-cell non-Hodgkin lymphoma, accounting for approximately 20 to 30% of global cases. The leukemic phase is a rare presentation. It is often associated with high-risk characteristics, high tumor burden, slow progression, diagnostic challenges, and unfavorable prognosis. Risk stratification using the Follicular Lymphoma International Prognostic Index (FLIPI) is crucial, since individuals classified as high-risk (FLIPI ≥3) have aggressive disease with inferior progression-free and overall survival outcomes. We report a case of a 51-year-old woman with extensive lymphadenopathy and leukocytosis, subsequently diagnosed with the leukemic phase of follicular lymphoma, with a FLIPI score of 3. She was treated with bendamustine and rituximab (BR regimen), along with supportive prophylaxis and growth factor support. The patient tolerated six cycles of the BR regimen with clinical enhancement and hematological stability. This case report emphasizes the pivotal role of integrating timely diagnostic evaluation, accurate risk stratification, and early incorporation of risk-adapted contemporary chemoimmunotherapy approaches in the management of the leukemic phase of follicular lymphoma, collectively leading to favorable patient outcomes.

## Introduction

Follicular lymphoma (FL) is the second most prevalent subtype of non-Hodgkin lymphoma, representing about one-third of indolent lymphomas [1]. The disease commonly presents with widespread lymphadenopathy, bone marrow infiltration, and systemic symptoms. However, leukemic-phase involvement, characterized by dissemination of malignant lymphoid cells into the peripheral blood, occurs in fewer than 10% of patients [2]. This rare presentation may produce leukemia-like hematological findings and is often associated with advanced Ann Arbor stage, high tumor burden, and adverse prognostic features.

The Follicular Lymphoma International Prognostic Index (FLIPI) utilizes five clinical parameters--age, Ann Arbor stage, hemoglobin level, the number of nodal sites involved, and serum lactate dehydrogenase (LDH) level--to categorize patients into risk categories [3]. Patients exhibiting a FLIPI score of 3-5 are classified as high-risk and have significantly reduced overall survival [4,5].

Although the illness is deemed incurable with conventional therapy, advancements in chemoimmunotherapy have improved outcomes. Bendamustine combined with rituximab (BR) has shown superior progression-free survival and better tolerability relative to rituximab plus cyclophosphamide, doxorubicin, vincristine, and prednisone (R-CHOP) or rituximab plus cyclophosphamide, vincristine, and prednisone (R-CVP) regimens, establishing it as a widely accepted first-line treatment regimen [5,6]. Nonetheless, disease relapse and histologic transformations are common and remain important clinical challenges, necessitating long-term surveillance and follow-up [2].

We report the case of a 51-year-old woman diagnosed with leukemic-phase follicular lymphoma, classified as high-risk according to the Follicular Lymphoma International Prognostic Index, who was effectively treated with the BR regimen.

## Case presentation

A 51-year-old woman presented to our tertiary care center in Chennai, Tamil Nadu, in November 2024, with complaints of progressively enlarging swelling in the right cervical region for four months, later involving bilateral axillary lymph nodes. She also reported increased fatigue, with no history of fever, significant weight loss, night sweats, cough, hemoptysis, or bone pain. Initial fine-needle aspiration cytology (FNAC) from the cervical swelling suggested reactive lymphadenitis. However, owing to persistent lymphadenopathy, she was referred for further evaluation. She was a known case of bronchial asthma on inhaler therapy and had a past surgical history of two lower-segment cesarean sections. No significant family history was noted. On examination, pallor was present, along with multiple firms, non-tender cervical and axillary lymph nodes, the largest measuring 3 × 2 cm. Vital signs were stable, and systemic examination was unremarkable, with no clinically evident hepatosplenomegaly.

Initial laboratory investigations revealed microcytic hypochromic anemia, with marked leukocytosis. The total leukocyte count was 112530 cells/cumm, with the presence of 91% circulating atypical lymphoid cells, morphologically suspicious for neoplastic lymphoid proliferation, while platelet counts were within normal limits (Table 1). Renal and liver function tests were normal. Serological tests for HIV, hepatitis B surface antigen (HBsAg), and hepatitis C virus (HCV) were negative. The combination of generalized lymphadenopathy, marked leukocytosis, and circulating atypical lymphoid cells raised suspicion for an underlying lymphoproliferative malignancy.

**Table 1:** Serial complete blood count profile of the patient during the clinical course. MCV: mean corpuscular volume, MCH: mean corpuscular hemoglobin, PCV: packed cell volume.

Laboratory test	Reference ranges	Day 11	Day 20	Day 184
Hemoglobin	12-15 gm/dl	8.9	8.8	10.8
Total count	4000-11000 cells/cumm	112530	13520	4710
Polymorphs	45-70%	08	91.1	64.6
Lymphocytes	25-40%	Atypical lymphoid cells (91%)	6.5	14.2
Eosinophils	1-6%	0	0	14.0
Monocytes	2-10%	01	1.6	5.5
Basophils	0-1%	0	0.1	1.3
RBC count	3.8-4.8 million/cumm	3.89	3.61	3.90
Platelets	1.5-4.5 lakhs/cumm	2.46	2.07	2.20
MCV	86-101 fl	79.7	81.2	84.6
MCH	27-33 pg	22.9	24.4	27.7
PCV	36-46%	31.0	29.3	33.0

Positron emission tomography-computed tomography (PET-CT) scan demonstrated widespread metabolically active lymphadenopathy involving bilateral cervical, supraclavicular, axillary (largest measuring 33 mm × 21 mm size, standardized uptake value (SUV) max 13.41), brachial, mediastinal, hilar, abdominal, and pelvic regions, suggestive of disseminated disease (Figure 1). She underwent excisional biopsy of right level Ib and level II cervical lymph node, which confirmed the diagnosis of follicular lymphoma (Figures 2, 3). Sections from the lymph node showed a completely effaced architecture, with the entire lymph node replaced by closely packed follicles of varying sizes (Figure 2A). Follicles were composed of monomorphous population of atypical lymphoid cells that were small, showed clumped nuclear chromatin, irregular cleaved nucleus, and scant cytoplasm (Figure 2B). Centroblasts with prominent nucleoli and abundant cytoplasm were noted 6/high power field (HPF) (Figure 3). This histomorphology was in favor of low-grade follicular lymphoma.

**Figure 1:**
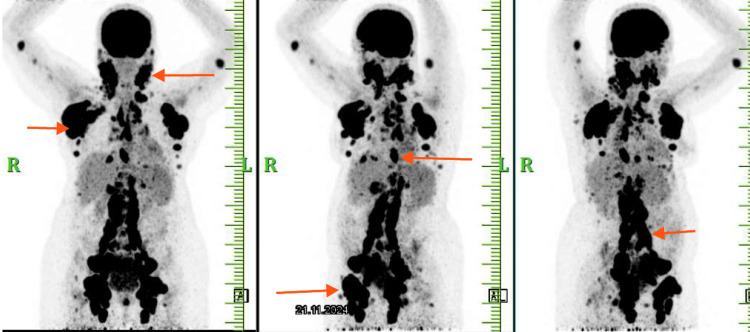
Baseline 18F-FDG PET-CT, done on day 7, demonstrating multiple FDG-avid lymphadenopathy (arrows). 18F-FDG PET-CT: 18F-fluorodeoxyglucose positron emission tomography-computed tomography.

**Figure 2:**
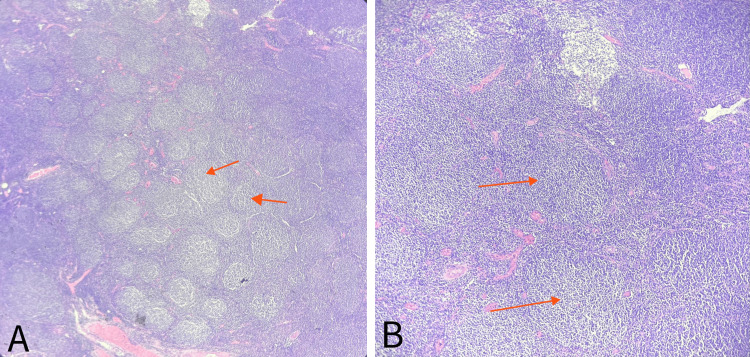
Hematoxylin and eosin (H&E) stained section showing closely packed follicles (arrows) composed of monomorphic population of atypical neoplastic lymphocytes. (A) Lymph node biopsy H&E stain (10× magnification) shows closely packed follicles of varying sizes. (B) Lymph node biopsy H&E stain (20× magnification) shows monomorphic population of atypical small lymphoid cells with irregular cleaved nucleus.

**Figure 3:**
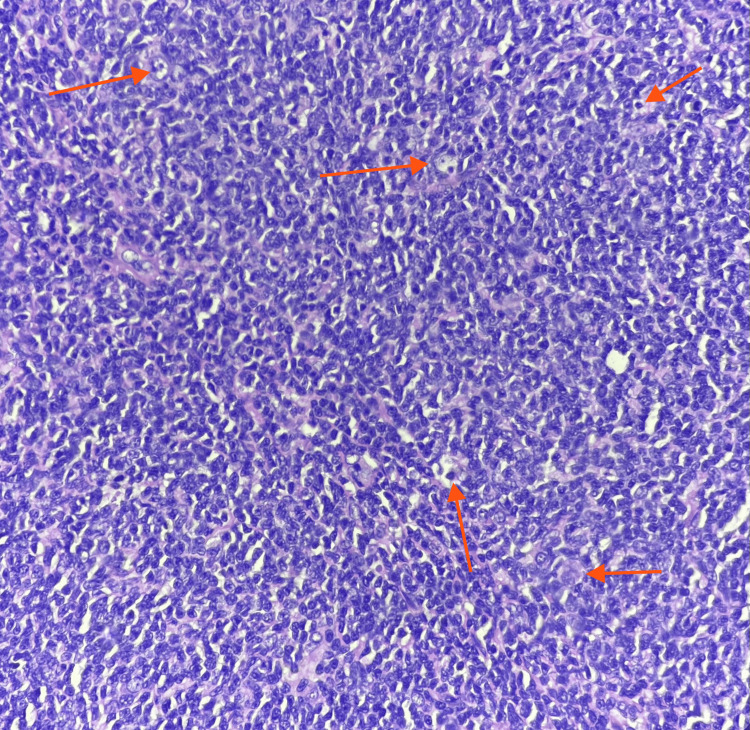
Lymph node biopsy, H&E stained section (40× magnification) showing the presence of centroblasts (6/HPF). HPF: high power field, H&E: hematoxylin and eosin.

Bone marrow aspirate (Figure 4) and biopsy (Figure 5) revealed infiltration by neoplastic lymphoid cells suggestive of marrow involvement by lymphoma. Flow cytometry immunophenotyping of peripheral blood was performed using a chronic lymphoproliferative disorder (CLPD) panel. Approximately 92% of the lymphoid cells comprised a clonal B-cell population expressing CD19, CD20, CD10, CD38, and kappa light-chain restriction, while negative for lambda, CD5 and CD23. These findings were consistent with the leukemic phase of follicular lymphoma and helped exclude other small B-cell lymphoproliferative disorders such as mantle cell lymphoma and chronic lymphocytic leukemia (CLL). Cytogenetic analysis showed a normal female karyotype (46, XX). Serum beta-2 microglobulin level was elevated at 2060 ng/mL, indicating a higher tumor burden.

**Figure 4:**
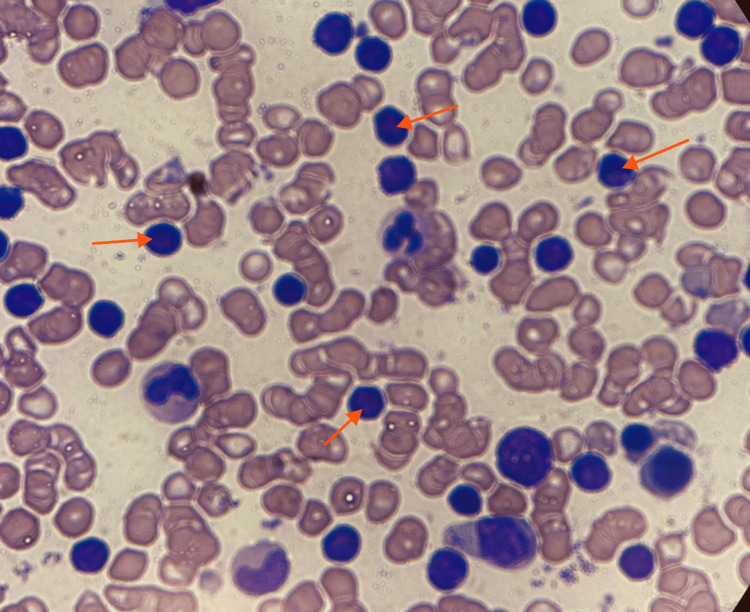
May-Grünwald-Giemsa-stained section of bone marrow aspirate (100× magnification) showing atypical neoplastic lymphoid cells (arrows).

**Figure 5:**
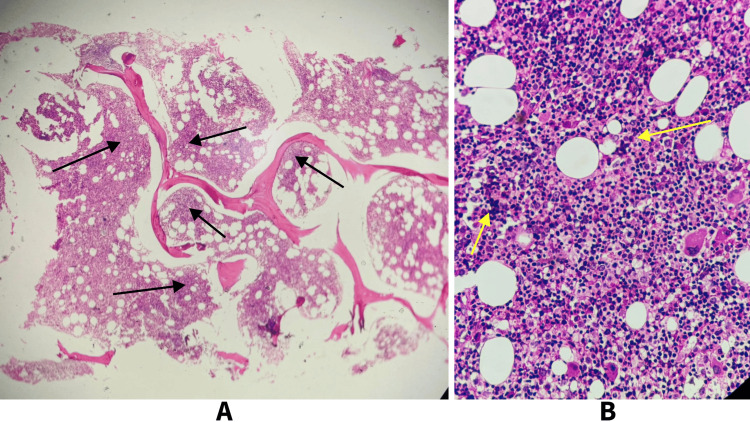
Bone marrow biopsy. (A) H&E stained section of bone marrow biopsy (10× magnification) showing para-trabecular lymphoid aggregates (arrows). (B) H&E stained section of bone marrow biopsy (40× magnification) showing a monomorphic population of atypical neoplastic lymphocytes in the para-trabecular region (arrows). H&E: hematoxylin and eosin.

Based on the clinical and laboratory findings, the patient fulfilled three FLIPI criteria: Ann Arbor stage IV disease, involvement of more than four nodal sites, and hemoglobin level <12 g/dL, resulting in a FLIPI score of 3 and categorizing her as high risk. The patient was initiated on bendamustine and rituximab (BR regimen) chemotherapy, followed by rituximab maintenance therapy. She completed six cycles of chemotherapy with good tolerance. Follow-up evaluation demonstrated clinical and hematological improvement, with normalization of total leukocyte counts. The patient remained clinically stable throughout each cycle and was discharged with advice on infection prevention, adequate nutritional support, and regular follow-up with serial complete blood count monitoring (Table 1). Follow-up PET-CT confirmed complete metabolic resolution of previously noted lymph nodes (Figure 6). A chronological summary of the patient’s clinical presentation, diagnostic evaluation, treatment course, and follow-up is provided in Table 2.

**Figure 6:**
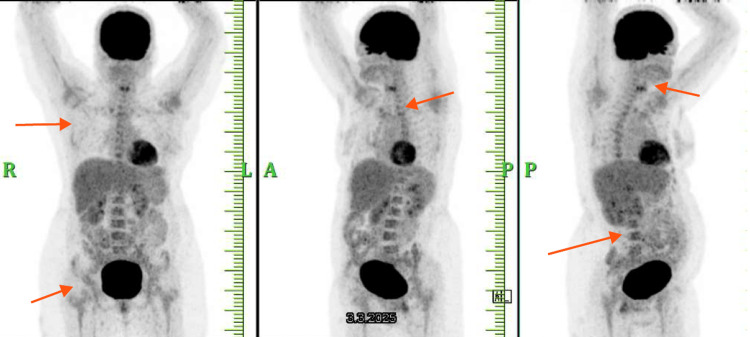
18F-FDG PET-CT, done on day 110, showing complete metabolic resolution of previously noted FDG-avid lymphadenopathy (Deauville Score 2). 18F-FDG PET-CT: 18F-fluorodeoxyglucose positron emission tomography-computed tomography. Deauville Score [7].

**Table 2:** Clinical timeline of the patient. PET-CT: positron emission tomography-computed tomography, FLIPI: Follicular Lymphoma International Prognostic Index, FDG: fluorodeoxyglucose.

Date	Event	Outcome
Four months before presentation	Onset of right cervical swelling	Progressive cervical lymphadenopathy
Day 0	Day of presentation	Bone marrow aspiration and biopsy done
Day 7	PET-CT scan	Disseminated metabolically active lymphadenopathy suggestive of systemic disease
Day 12	Excisional cervical lymph node biopsy	Histopathological confirmation of follicular lymphoma
Day 14	Bone marrow aspirate and flow cytometry	Leukemic phase follicular lymphoma confirmed; FLIPI score 3 (high risk)
Day 19	Serum beta-2 microglobulin assessment	Elevated (2060 ng/mL), indicating high tumor burden
Day 19	Initiation of bendamustine-rituximab (BR regimen)	Commencement of first-line chemoimmunotherapy
Day 110	Follow-up PET-CT scan	Complete metabolic resolution of previously FDG-avid lymph nodes.
Day 184	Completion of six cycles of BR chemotherapy	Clinical stability with good treatment tolerance
Day 184	Follow-up complete blood count	Normalization of leukocyte count
Post-chemotherapy	Rituximab maintenance therapy initiated	Sustained disease control with stable clinical status
Ongoing	Regular hematology-oncology follow-up	Monitoring for relapse, treatment response, and therapy-related complications

## Discussion

Leukemic phase of follicular lymphoma is an uncommon presentation and often poses significant diagnostic challenges due to the presence of circulating atypical lymphoid cells that can mimic other lymphoproliferative disorders, such as chronic lymphocytic leukemia (CLL) and mantle cell lymphoma. Therefore, definitive diagnosis requires a multimodal approach integrating histopathology, immunophenotyping, and flow cytometry and radiological assessment [2].

In the presented case, PET-CT imaging, excisional lymph node biopsy, and flow cytometric analysis collectively aided accurate diagnosis. The neoplastic cells demonstrated a characteristic immunophenotype (CD10+, CD19+, CD20+), consistent with follicular lymphoma involving peripheral blood and bone marrow. Although the t(14;18) translocation is frequently associated with follicular lymphoma, its absence in this case highlights the biological heterogeneity of the disease [8,9].

Bendamustine-rituximab (BR regimen) is an established first-line treatment option for indolent non-Hodgkin lymphomas. The BRIGHT study and the StiL NHL1 trial demonstrated superior progression-free survival and a favorable toxicity profile with the BR regimen compared to R-CHOP and R-CVP regimens. Long-term follow-up data further support durable responses, with median progression-free survival approaching 69 months [5,6].

Despite therapeutic advances, follicular lymphoma remains a chronic disease, incurable but manageable, characterized by repeated relapses. Patients with high-risk disease, defined by a FLIPI score ≥3, have less favorable long-term outcomes, necessitating close surveillance and maintenance strategies. Progression of disease within 24 months of treatment initiation (POD24) has emerged as a critical prognostic marker associated with inferior overall survival [4]. Patients with POD24 exhibit higher tumor burden, shorter progression-free interval, aggressive biological behavior, and a greater likelihood of transformation to aggressive lymphoma, such as diffuse large B-cell lymphoma [10].

In contrast, patients without POD24 tend to have more favorable long-term survival outcomes. The PRIMA trial demonstrated that Rituximab maintenance therapy for two years significantly improves progression-free survival in patients with high tumor burden [11].

Supportive care plays a crucial role in patient management, particularly in patients receiving chemoimmunotherapy. Immunocompromised patients are at increased risk of opportunistic infections; therefore, prophylactic measures, including infection surveillance, appropriate antimicrobial prophylaxis when indicated, along with growth factor support, nutritional support, and regular hematological monitoring, are essential to improve tolerability and reduce treatment-related morbidity [12]. In this case report, the patient tolerated treatment well, with no major infectious complications. This likely reflects the role of standard supportive care measures in reducing treatment-related morbidity in patients receiving chemoimmunotherapy.

This case reinforces the importance of comprehensive and multimodal diagnostic evaluation, appropriate risk stratification, and evidence-based chemoimmunotherapy in the management of leukemic phase of follicular lymphoma. Early recognition and timely intervention can lead to favorable clinical outcomes and improved quality of life, even in patients presenting with high-risk disease.

## Conclusions

The leukemic phase of follicular lymphoma is an uncommon but clinically significant presentation of an otherwise indolent lymphoma. Accurate diagnosis using histopathology, flow cytometry, and immunophenotyping, with appropriate risk stratification using FLIPI score, is essential for optimal disease management. Early initiation of effective chemoimmunotherapy, such as a bendamustine-rituximab regimen, can achieve a favorable clinical and hematological response. Supportive therapy, including infection prevention and growth factor administration, when indicated, plays an important role in reducing treatment-related morbidity in patients receiving chemoimmunotherapy. Despite a cautious long-term prognosis for high-risk individuals, advances in contemporary therapy may provide durable remission and sustained clinical stability.
